# Evaluating Single-Nucleotide Polymorphisms in Inflammasome Proteins and Serum Levels of IL-18 and IL-1β in Kidney Interstitial Damage in Anti-Neutrophilic Cytoplasmic Antibody-Associated Vasculitis

**DOI:** 10.3390/ijms25126479

**Published:** 2024-06-12

**Authors:** Laura Martinez Valenzuela, Anna Vidal-Alabró, Belén Rubio, Paula Antón-Pàmpols, Francisco Gómez-Preciado, Xavier Fulladosa, Josep Maria Cruzado, Juan Torras, Nuria Lloberas, Juliana Draibe

**Affiliations:** 1 Nephrology Department, Bellvitge University Hospital, 08907 L’Hospitalet de Llobregat, Spain; lmartinezv@bellvitgehospital.cat (L.M.V.); brubio@bellvitgehospital.cat (B.R.); pantonp.germanstrias@gencat.cat (P.A.-P.); fgomezp@bellvitgehospital.cat (F.G.-P.); xfulladosa@bellvitgehospital.cat (X.F.); jmcruzado@bellvitgehospital.cat (J.M.C.); jbordignon@bellvitgehospital.cat (J.D.); 2 Experimental Nephrology Laboratory, Institut d’Investigació Biomèdica de Bellvitge (IDIBELL), 08907 L’Hospitalet de Llobregat, Spain; annavidal@ub.edu (A.V.-A.); nlloberas@idibell.cat (N.L.); 3 Faculty of Medicine, Bellvitge Campus, University of Barcelona, 08907 L’Hospitalet de Llobregat, Spain

**Keywords:** inflammasome, ANCA-associated vasculitis, interleukin-18 (IL-18), single-nucleotide polymorphisms (SNPs), biomarkers

## Abstract

The inflammasome regulates the innate inflammatory response and is involved in autoimmune diseases. In this study, we explored the levels of IL-18 and IL-1β in serum and urine and the influence of various single-nucleotide polymorphisms (SNPs) on kidney lesions at diagnosis in patients with ANCA-associated vasculitis (AAV) and their clinical outcomes. Ninety-two patients with renal AAV were recruited, and blood and urine were collected at diagnosis. Serum and urine cytokine levels were measured by ELISA. DNA was extracted and genotyped using TaqMan assays for SNPs in several inflammasome genes. Lower serum IL-18 (*p* = 0.049) and the *IL-18* rs187238 G-carrier genotype (*p* = 0.042) were associated with severe fibrosis. The *IL-18* rs1946518 TT genotype was associated with an increased risk of relapse (*p* = 0.05), whereas GG was related to better renal outcomes (*p* = 0.031). The rs187238 GG genotype was identified as a risk factor for mortality within the first year after AAV diagnosis, independent of the requirement for dialysis or lung involvement (*p* = 0.013). We suggest that decreased cytokine levels could be a surrogate marker of scarring and chronicity of the renal lesions, together with the rs187238 GG genotype. If our results are validated, the rs1946518 TT genotype predicts the risk of relapse and renal outcomes during follow-up.

## 1. Introduction

Inflammasomes are large protein complexes that activate innate immunity in response to inflammatory signals related to infectious agents and cell damage. The upstream sensor molecule of the inflammasome complex triggers the activation of an effector protein (caspase-1 is the most studied), leading to the release of pro-inflammatory cytokines, such as interleukin (IL)-18 and IL-1β, and pyroptotic cell death [1]. The nucleotide-binding oligomerization domain (NOD)-like receptor family pyrin domain-containing-3 (NLRP3) inflammasome is the most thoroughly investigated. Sterile inflammation activates the NLRP3 inflammasome, in turn regulating IL-1β production in macrophages. The effector cytokines of the inflammasome pathway, IL-18, and IL-1β, trigger the release of other cytokines such as IL-1α, IL-6, and tumor necrosis factor (TNF)-α, among other factors that control proliferation and the phenotype of immune cells [2]. Aberrant inflammasome assembly and activation, which has been associated with the presence of certain single-nucleotide polymorphisms (SNPs) in inflammasome genes, may contribute to the development of autoimmune diseases, such as rheumatoid arthritis [3,4], systemic lupus erythematosus [5], or systemic sclerosis [6].

Anti-neutrophilic cytoplasmic antibody (ANCA)-associated vasculitis (AAV) is an autoimmune multisystemic disease affecting small-sized vessels. Renal involvement is frequent, with the most common form of kidney disease being rapidly progressive pauci-immune glomerulonephritis [7]. However, clinical presentation as isolated acute interstitial nephritis in the absence of glomerular lesions is possible [8]. Renal outcome prediction in AAV is often performed using scales that consider the extent and type of glomerular lesions, such as crescents or scars [9]. In recent years, the importance of grading the interstitial lesions present in the diagnostic kidney biopsy has increased, as some studies demonstrate their prognostic value [10].

Some studies investigated the activation and dysregulation of the inflammasome pathways in AAV. Hewins et al. found that in patients with renal AAV, podocytes, myofibroblasts, distal tubular epithelium, and infiltrating macrophages all stained positively for IL-18. In the same study, the authors also observed IL-18 primed superoxide production by ANCA-activated neutrophils in vitro [11]. Tashiro et al. studied the expression of diverse inflammasome components, cytokines, and molecules involved in sterile inflammation in a set of 28 kidney biopsies from AAV patients. They found a significant correlation between the IL-1β expression and the severity of tubulointerstitial damage. Furthermore, they observed an increased expression of toll-like receptor (TLR)-4—an activator of the innate immune response—in cells infiltrating the tubulointerstitium, while there was an increased expression of NLRP3 in the macrophages in severely infiltrated interstitial areas. The expression of these molecules also correlated with the severity of the lesions found in the interstitium. Altogether, the authors hypothesized that sterile inflammation stimulates macrophages via TLR4 and promotes NLRP3 inflammasome-dependent processing and the release of IL-1β in AAV [12]. In the same vein, Wang et al. found that the expression of different inflammasome molecules—NOD2, NLRP3, and NOD-like receptor family caspase activation and recruitment domain (CARD) containing 5 (NLRC5)—was significantly higher compared to the expression found in other glomerulonephritides and healthy controls, especially when co-localizing with podocytes and infiltrating inflammatory cells. Interestingly, glomerular expression of NOD2 was higher in biopsies classified as crescentic according to the Berden Classification compared to the rest, and this correlated with proteinuria and serum creatinine levels [13].

Genotypes of SNPs located in different genes involved in the function of the immune system have been evaluated as risk factors for susceptibility and clinical outcomes of AAV. Several authors studied the association of certain SNPs in the human leukocyte antigen (HLA) regions and found differences in frequency according to ANCA specificity (myeloperoxidase (MPO) or proteinase-3 (PR3)) [14,15]. Casal Moura et al. found an association of rs351111, located in the PR3 gene, with a higher frequency of severe relapses in a cohort of 188 patients [16]. Certain *TLR9*, *protein tyrosine phosphatase non-receptor type 22* (*PTPN22*), and *IL2 receptor-A* SNPs involved in lymphocyte activation pathways have also been associated with higher disease susceptibility, suggesting their pathophysiologic role [17,18,19]. Unfortunately, the prevalence of SNPs of inflammasome-related genes in AAV has not been studied extensively.

In this study, our aim was to describe the frequency of a set of SNPs in inflammasome genes and the concentration of the final products of the inflammasome pathway, IL-18, and IL-1 β, in serum and urine in a cohort of patients affected with AAV and their association with clinical features and disease outcomes. We focused on the correlation of these SNPs with lesions found in kidney biopsies since a differential expression of inflammasome-associated molecules depending on the severity of the lesions has been reported, as well as the prognostic implications of renal lesions on the evolution of kidney function.

## 2. Results

### 2.1. Characteristics of the Study Population

Ninety-two patients diagnosed with AAV with kidney involvement were recruited. Table 1 shows the baseline characteristics of the patients.

### 2.2. Serum and Urinary IL-18 and IL-1β Levels

The samples were obtained at the time of recruitment. Of the 92 patients recruited and sampled, 35 were at the time of diagnosis. Of those, 26 also had urine samples available. The remaining patients were recruited during the remission phase, so they were not included in the cytokine analysis.

We examined the concentration of cytokines focusing on the different lesions observed in the diagnostic kidney biopsy. Notably, both serum IL-18 (sIL-18) and IL-1β (sIL-1β) concentrations were higher in patients with grade 0 or 1 interstitial fibrosis compared to those with grade 2 or 3 fibrosis. Additionally, sIL-1β was significantly higher in patients with grade 0 or 1 compared to those with grade 2 or 3 atrophy. Similarly, a trend towards higher sIL-18 was observed in patients with grade 0–1 atrophy (see Table 2).

Regarding the glomerular lesions, patients classified as sclerotic according to the Berden Classification presented lower levels of sIL-18 (75.82 ± 65.90 pg/mL vs. 458.47 ± 507.29 pg/mL *p* = 0.032) and sIL-1β (0.19 ± 0.32 pg/mL vs. 2.11 ± 6.02 pg/mL *p* = 0.028) compared to patients included in the other categories (see Supplementary Table S1). 

There were no statistically significant differences in cytokine levels in the elderly compared to younger patients, as well as between gender and ANCA type. Patients with PR3 ANCA tended to have higher sIL-18 compared to patients with MPO ANCA, although the differences did not reach statistical significance (*p* = 0.08) (Supplementary Table S1). We found no differences in the urine levels of IL-1β across the different variables evaluated.

### 2.3. Allele and Genotype Distribution

The genotype frequencies observed in the population are in agreement with the expected frequencies according to the Hardy–Weinberg equilibrium. The minor allele frequency (MAF) of the SNPs was similar to the MAF reported in the 1000 genomes project in the European population (see Table 3).

### 2.4. IL-18 SNPs, sIL-18 Levels, and Lesions in the Kidney Biopsy

In our study results, we have focused on elucidating the implications of IL-18 SNPs, as they have yielded the most intriguing and biologically plausible outcomes. No statistically significant associations were found between the remaining polymorphisms studied and the characteristics of renal biopsy lesions, analytical parameters, or clinical outcomes of the patients (Supplementary Table S2).

### 2.5. The IL-18 rs187238 G Allele Is Associated with Lower sIL-18, More Severe Fibrosis, and Higher Mortality

**Figure 1:**
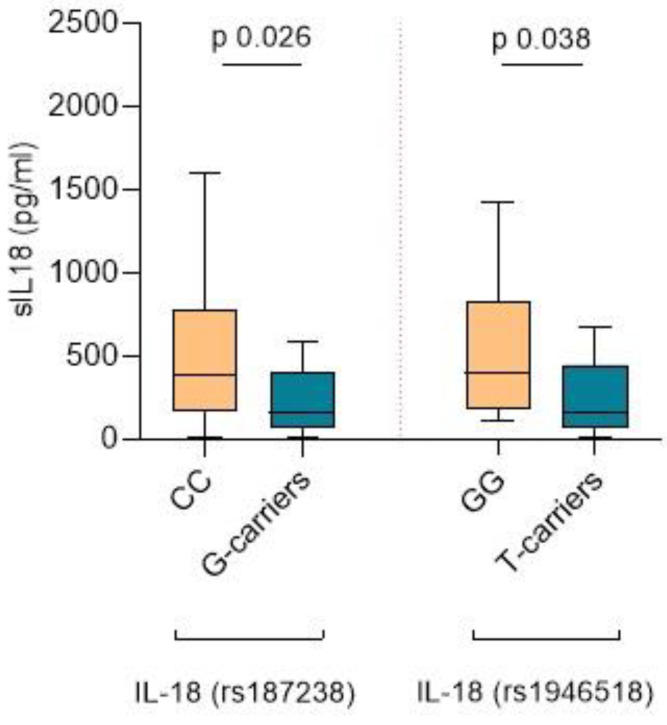
Levels of IL-18 in serum according to the rs187238 and rs1946518 genotypes.

Interestingly, the *IL-18* rs187238 G allele was associated with lower sIL-18 levels. Patients with the G-carrier genotype exhibited significantly lower sIL-18 levels compared to CC-carriers (145.5 pg/mL, IQR 60.2–514.5 vs. 384.8 pg/mL, IQR 177.4–830.2, *p* = 0.026) (Figure 1). However, no significant differences were found in sIL-18 levels in patients with the CG or GG genotype.

Our results revealed a distinct pattern of interstitial kidney lesions based on the genotype of *IL-18* rs187238, with the most severe interstitial lesions being linked to the G allele. Specifically, the GG genotype showed an association with an increased risk of severe fibrosis (OR 7.87, 95% CI 1.092–56.778, *p* = 0.019). We did not find differences in the glomerular lesions present in the kidney biopsy (according to the Berden Classification [9]) across the different genotypes of rs187238.

In the analysis of analytical parameters, C-carrier patients showed no discernible differences in renal function, proteinuria, or C-reactive protein (CRP) levels upon diagnosis (see Table 4).

**Figure 2:**
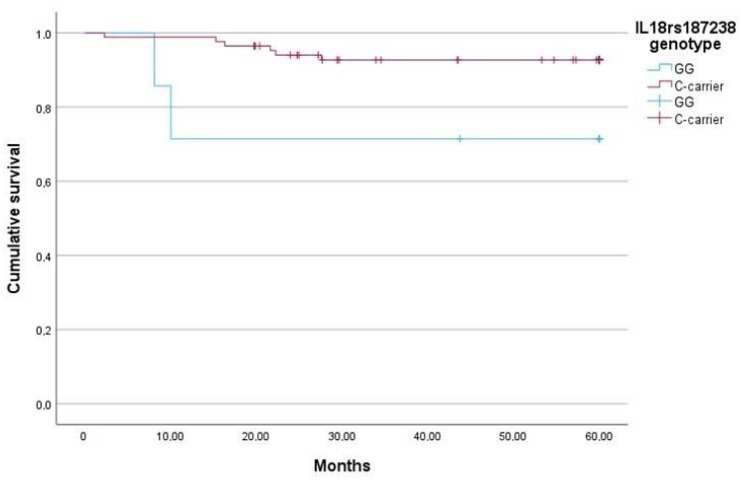
Kaplan–Meier survival curve according to the *IL-18* rs187238 genotype.

In addition, *IL-18* rs187238 GG was identified as a risk factor for mortality within the first year after the diagnosis of AAV, according to the results of the univariate logistic regression analysis (Exp(B) 33.6, 95% CI 2.58–436.55, *p* = 0.007). The increased risk was independent of the requirement of renal replacement therapy (RRT) or lung involvement in the multivariate analysis (Exp(B) 27.031, 95% CI 2.005–364.4, *p* = 0.013). A Kaplan–Meier analysis was performed to assess the survival outcomes in our cohort of patients diagnosed with AAV (Figure 2). The median survival time for patients with the IL-18 rs187238 GG genotype was 99.576 months (95% CI 47.63–151.52), whereas for C-carriers, it was 368.67 months (95% CI 317.56–419.77). After 1 year, the estimated survival rates were 71.4% for patients with the GG genotype and 98.8% for C-carrier patients, with a log-rank test confirming the statistical significance of the differences (*p* < 0.001). After 3 and 5 years, the estimated survival rates were 71.4% for the GG genotype and 92.9% for C-carriers. The log-rank test revealed a statistically significant difference in survival between the two groups (*p* = 0.033) in both cases. We did not observe differences in renal survival or incidence of relapse according to the *IL-18* rs187238 genotype. 

### 2.6. The IL-18 rs1946518 T Allele Is Associated with Lower sIL-18, Lower eGFR Levels during Follow-Up, and a Higher Risk of Relapse within the First Year after Diagnosis

The different *IL-18* rs1946518 genotypes were also associated with variations in sIL-18 levels. Patients exhibiting the T-carrier genotype had lower levels of sIL-18 compared to the GG genotype (162.05 pg/mL, IQR 67.63–449.56 pg/mL vs. 401.85 pg/mL, IQR 177.37–830.19 pg/mL, *p* = 0.038) (Figure 1). However, no differences were observed in the severity of the interstitial renal lesions or in the glomerular lesions present in the kidney biopsy (according to the Berden Classification).

With regard to the review of analytical parameters, patients with the T-carrier genotype did not exhibit differences in renal function, proteinuria, or CRP levels at the time of diagnosis (Table 5).

In terms of the progression of kidney function over time, it was noted that patients with the T-carrier genotype for *IL-18* rs1946518 exhibited lower estimated glomerular filtration rate (eGFR) levels at 6, 12, and 24 months post-diagnosis. However, statistical significance was only reached at 24 months (38.98 ± 18.21 mL/min vs. 52.58 ± 27.61 mL/min, *p* = 0.031). We then shifted our focus to analyzing the risk of relapse. In the binary logistic regression univariate analysis, the rs1946518 TT genotype was associated with a higher risk of relapse during the first year after diagnosis (Exp(B) 3.8, 95% CI 1.087–13.289, *p* = 0.037). However, we found no significant differences in mortality risk based on the rs1946518 genotype.

### 2.7. Association of SNPs and Organ Involvement in AAV

We investigated the association of certain SNPs with an increased risk of organ damage in AAV. Our data revealed that patients with the NLRP1 rs878329 CC genotype carried a higher risk for ENT involvement (OR 3.86, 95% CI 1.034–14.481, *p* = 0.05). Additionally, the CARD8 rs2043211 TT genotype was identified as a risk factor for neurological involvement in our patient cohort (OR 6.041, 95% CI 1.413–25.818, *p* = 0.024), along with the patients presenting the CASP1 rs530537 TT genotype (OR 9.33, 95% CI 2.241–38.867, *p* = 0.001). Conversely, no increased risk of organ damage was found to be associated with the genotypes of the remaining SNPs evaluated.

## 3. Discussion

The inflammasome system plays a crucial role in the activity of adaptive immunity by regulating the production of downstream inflammatory cytokines, such as IL-1β and IL-18 [20]. Upon activation, inflammasome triggers a cascade of inflammatory responses, leading to the release of additional pro-inflammatory cytokines and the polarization of the T-lymphocyte response towards a T-helper (Th)-1 phenotype [21], which is predominant in AAV-associated glomerulonephritis [22]. In this study, our primary aim was to investigate the influence of specific SNPs in inflammasome-related genes and their impact on disease progression. Additionally, we aimed to examine differences in the serum levels of downstream products of the inflammasome, focusing on the lesions identified in the diagnostic kidney biopsy and their impact on disease progression. By elucidating these factors, we sought to gain insights into the mechanisms underlying AAV pathogenesis and identify potential targets for therapeutic intervention.

In our study, patients with chronic interstitial damage, characterized by fibrosis and atrophy, exhibited decreased levels of IL-18 and IL-1β cytokines in serum compared to patients with mild chronic lesions, who demonstrated the highest levels. IL-1β, acting as a downstream mediator in the development of immune-mediated glomerulonephritides, plays a pivotal role in the inflammatory cascade [23]. While glomerular cells themselves do not directly produce IL-1β, they exhibit a strong responsiveness to this cytokine [24]. Studies on IL-1β knock-out mice have revealed an attenuated phenotype of crescentic glomerulonephritis in response to anti-mouse glomerular basement membrane (GBM) globulin administration [25]. Furthermore, several authors have described that different kidney cells, including mesangial and proximal tubular cells, respond to IL-1β by inducing fibrosis. For instance, Pawluczyk et al. demonstrated an increased in vitro production of fibronectin by mesangial cells exposed to IL-1β [26], while IL-1β was found to suppress proliferation and enhance fibronectin production in human proximal tubular cells. Despite several studies on serum IL-1β levels in autoimmune diseases and cancer, significant results supporting their role as a biomarker have yet to be consistently found [27,28,29,30]. Nevertheless, we observed differences in serum IL-1β levels, with a gradient from higher to lower levels in patients presenting with less to more chronic tubular atrophy, suggesting a potential role for IL-1β in the pathogenesis of chronic kidney disease.

Elevated IL-18 serum levels have been previously reported in AAV patients by Hultgren et al. when compared to healthy controls [31]. Additionally, Liu et al. found elevated levels of sIL-18 in active AAV compared to patients in remission and healthy controls, with a positive correlation observed with the Birmingham Vasculitis Activity Score (BVAS) [32]. Notably, neither of these studies investigated the association of these elevated levels with histopathologic kidney lesions in AAV. Nonetheless, the association between IL-18 levels and kidney histopathology has been studied in other immune-mediated nephropathies. For instance, Shi et al. described higher levels of sIL-18 in patients with IgA nephropathy (IgAN) and a higher interstitial damage score, which is based on the sum of atrophy, fibrosis, and interstitial infiltrate [33]. However, while our study focuses on the relationship between sIL-18 levels and the severity of specific interstitial lesions, Shi et al.’s study examines the relationship between sIL-18 and a general interstitial damage score that does not differentiate between different types of lesions. This approach may lead to biased conclusions, given the potential differences in sIL-18 levels in patients with chronic lesions, such as fibrosis and atrophy, compared to acute lesions, such as interstitial inflammatory infiltrate. In our study, we observed the highest levels of cytokines in the absence of chronic interstitial lesions, suggesting that the decrease in serum concentration could serve as a marker of evolution towards fibrosis and chronicity. We propose that following the initial renal injury, inflammasome pathways are initiated, leading to the secretion of cytokines. Subsequently, reparative processes are initiated, and pro-fibrotic cells become activated, resulting in a decrease in cytokine levels [34].

The *IL-18* rs187238 SNP, located in the promoter region, has been shown to alter the binding site of the histone H4 gene-specific transcription factor-1 (H4TF-1) nuclear factor [35]. In our study, we found that the GG genotype was associated with a higher severity of interstitial fibrosis in the diagnostic kidney biopsy and lower sIL-18 levels. As expected, our findings are in line with those of other authors who also reported higher sIL-18 levels in patients with the minority allele in homozygosis and kidney graft acute rejection [36] or IgAN [37]. Giedraitis et al. have described that polymorphisms in the IL-18 promoter affect its activity, but this effect may vary according to the cell type and the local cytokine environment. Peripheral blood mononuclear cells (PBMCs) from healthy controls with different genotypes respond to lipopolysaccharide (LPS) by releasing variable amounts of IL-18 [38]. Further research is warranted to elucidate the specific mechanisms underlying these observations and their implications for disease pathogenesis and management.

We also assessed the rs1946518 SNP, located in the IL-18 gene. Our findings revealed that patients with the GG genotype exhibited significantly higher sIL-18 levels compared to T-carriers and TT-homozygotes. However, we did not observe differences in the extent of tubulointerstitial lesions based on the rs1946518 genotype. This finding contrasts with those of other authors, such as Zhang et al., who did not find differences based on rs1946518 in a cohort of patients diagnosed with schizophrenia [39], nor did Pavlovna et al. in healthy controls [35]. The observation of higher sIL-18 levels in AAV patients with the GG genotype, but not in other populations, may underscore the interaction between the IL-18 genotype and the inflammatory environment in AAV. Consistent with our results, Yang et al. found no differences in the composite score of interstitial fibrosis and tubular atrophy in a cohort of IgAN patients based on the rs1946518 genotype [40]. These findings suggest that the impact of the rs1946518 SNP on sIL-18 levels and tubulointerstitial lesions may vary, depending on the underlying inflammatory condition and genetic background.

We sought to investigate the influence of the evaluated SNPs on the prognosis of patients with AAV. In our study, we observed a protective effect of the IL-18 rs1946518 GG genotype on kidney function during follow-up. This finding aligns with the results of Pawlus et al., who reported a 2.35 times higher risk of chronic rejection in kidney transplant patients with the complementary AA genotype [41]. The improved renal outcomes associated with the GG genotype may also be attributed to the higher risk of relapse observed in patients with the rs1946518 TT genotype. Furthermore, we identified the rs187238 GG genotype as a risk factor for mortality in our AAV cohort. Notably, while rs187238 GG is known as a risk factor for cardiovascular events [42], the cause of death among rs187238 GG patients in our cohort was mainly related to respiratory failure.

While certain SNP genotypes were associated with a higher susceptibility to specific organ involvement in AAV, in our study, we were unable to confirm such susceptibility to organ involvement in other autoimmune diseases through the literature review. Moreover, since all patients in our cohort presented with renal manifestations, we could not assess whether any of the studied polymorphisms elevate the risk of renal involvement in AAV.

We must highlight the novelty of our topic as one of the main strengths of our study. To the best of our knowledge, this is the first investigation into inflammasome-related gene SNPs in a vasculitis cohort. Furthermore, while studies on renal AAV typically focus on glomerular lesions, our study centers on investigating lesions in the tubulointerstitial area, providing valuable insights into a less explored aspect of the disease. Despite the rarity of ANCA vasculitis and the challenges in assembling large cohorts, we successfully recruited a significant number of patients. However, a major limitation of this study is the absence of a validation cohort to confirm the findings. Nevertheless, the results are supported by their biological plausibility and by studies in other autoimmune diseases. This study was conducted on patients from our geographical region, which may affect the applicability of the results to other areas and constitutes another limitation of our study.

Basic research studies play a crucial role in unraveling the underlying mechanisms, with the goal of identifying novel disease biomarkers and therapeutic targets. In recent years, there has been a growing focus on investigating the therapeutic potential of various inflammasome-targeting therapies [43,44]. We hypothesize that the blockade of the inflammasome complex could offer a promising therapeutic strategy for patients with AAV, potentially leading to improved treatment outcomes.

## 4. Methods

### 4.1. Study Population

All patients diagnosed with AAV with renal involvement who were under follow-up at our institution were eligible for this study. Each patient met the Chapel Hill Consensus criteria for small vessel vasculitis classification [45] and was in the acute disease phase, indicated by a BVAS score above 0 [46]. These patients were prospectively monitored at our institution and received standard immunosuppressive treatment. Induction therapy typically involved intravenous methylprednisolone pulses and/or plasmapheresis followed by rituximab and/or cyclophosphamide, while maintenance therapy included oral prednisone, mycophenolate mofetil, azathioprine, or rituximab. Patients with end-stage kidney disease, active non-AAV autoimmune conditions, active neoplasms, or infections were excluded from this study.

### 4.2. Clinical and Laboratory Data

Demographic, clinical, and analytical data were retrieved from electronic medical records, encompassing age, gender, date of diagnosis, treatments administered, ANCA titers, serum CRP, hematuria, proteinuria (protein-to-creatinine ratio, PCR), serum creatinine, eGFR, incidence of relapses, and patient and renal survival. Histopathological assessment was conducted by a specialized pathologist from our institution. Kidney biopsies were classified based on the presence of glomerular lesions according to the Berden Classification [9]. Tubulointerstitial lesions—infiltrate, atrophy, and fibrosis—were graded based on their extent: grade 0 (affecting <25%), grade 1 (affecting 25–50%), grade 2 (affecting 50–75%), and grade 3 (affecting >75%). Details regarding induction therapy, maintenance treatment, and patient and renal survival were also documented. 

### 4.3. Sampling, Genotyping Assays, and Cytokine Assessment

Peripheral blood and urine samples were collected from patients at the time of recruitment.

Genomic deoxyribonucleic acid (DNA) was extracted from the peripheral whole blood of patients using a Maxwell RSC^®^ instrument (Promega Corporation, Sydney, Australia). The DNA concentration and purity (260/280) were checked using a nano-drop spectrophotometer. Genotyping was performed using the TaqMan SNP genotyping assay (Applied Biosystems, Foster City, CA, USA) on the 7900HT Fast Real-time PCR System (Thermo Fisher Scientific, Waltham, MA, USA), following standard recommendations.

The concentration of IL-18 and IL-1β in serum and IL-1β in urine samples were measured using a commercial enzyme-linked immunosorbent assay (ELISA) kit according to the manufacturer’s instructions (Human Total IL-18/IL-1F4 Quantikine ELISA Kit, R&D systems^®^, Minneapolis, MN, USA, and IL-1β Human ELISA Kit, High Sensitivity, ThermoFisher^®^, Schwerte, Germany, respectively). 

### 4.4. Selection of SNPs

Initially, a literature search was conducted using the PubMed electronic database to identify SNPs in inflammasome-related genes associated with vasculitis. However, this search did not yield satisfactory results. Consequently, we extended our search to other autoimmune diseases using the query “[inflammasome polymorphisms & autoimmune disease]”. This search returned 53 papers published before 2020. After screening these papers, some were discarded for the following reasons: (a) their primary focus was on microRNA, (b) they were not available, or (c) they were reviews not strictly related to inflammasome. From the remaining studies, we compiled a list of polymorphisms in several inflammasome-related genes that were frequently mentioned in the literature.

To determine the most interesting polymorphisms for our study, we consulted the dbSNP database using the code for each polymorphism to find information on their prevalence in our population. Additionally, we performed another search in PubMed using the specific polymorphism number and autoimmune disease. We selected SNPs for our study based on two criteria: a minor allele frequency (MAF) over 10% among the European population (MAF > 0.1) and previous consideration in the literature related to autoimmunity (see Table 5). See Supplementary Table S3 for detailed results of the literature search.

### 4.5. Statistical Analysis

Data were analyzed using GraphPad Prism version 8.00 (GraphPad Software, Boston, MA, USA) and IBM SPSS Statistics Version 29.0 (IBM Corp., New York, NY, USA). The Gaussian distribution of variables was determined using the Kolmogorov–Smirnov test. The comparison of means for quantitative variables was performed using analysis of variance (ANOVA), Student’s *t*-test, or Mann–Whitney U tests. Chi-square tests were employed to compare the frequency of qualitative variables, and the corresponding odds ratio was calculated. The risk of presenting an outcome according to the different SNP genotypes was assessed by binary or multinomial logistic regression according to the nature of the categorical variable. *p*-values < 0.05 were considered statistically significant.

## 5. Conclusions

In summary, our study revealed notable associations between cytokine levels, genetic polymorphisms, and disease outcomes in patients with AAV. Higher serum levels of IL-18 and IL-1β were observed in patients with mild chronic lesions in the renal tubulointerstitium, which decreased in patients with higher degrees of chronicity. This suggests that the decrease in cytokine levels may serve as a surrogate marker of the scarring and chronification of renal lesions. Additionally, the rs187238 GG genotype was identified as a risk factor for the development of chronicity in renal lesions. Regarding the outcomes of the disease, the IL-18 rs1946518 GG genotype demonstrated a protective effect on kidney function during follow-up, potentially attributed to its association with a lower risk of relapse compared to the TT genotype. This highlights the importance of genetic factors influencing disease progression and treatment response in AAV patients.

## Figures and Tables

**Table 1:** Baseline characteristics of the population. IQR, interquartile range; MPO, myeloperoxidase; ANCAs, anti-neutrophil cytoplasm antibodies; SD, standard deviation; N/A, not assessed.

Gender (% male)	44.56
Median age (IQR) (years)	68 (60–76.75)
Organ involvement (%)	65.51
ANCA type (%MPO)	74.70
Median ANCA titer (IQR) (karbU/L)	13 (1.70–54)
Median serum creatinine IQR (µmol/L)	145 (104.51–180)
Mean eGFR ± SD (mL/min)	38.5 ± 18.63
Mean proteinuria ± SD (g/mol)	0.53 ± 0.60
Median serum CRP (IQR) mg/L	4 (1.40–11.92)
Berden Histopathologic Classification (% patients)	
Focal	18.47
Crescentic	26.08
Mixed	31.52
Sclerotic	11.95
N/A	10.86
Induction treatment (% patients)	
Methylprednisolone pulses	40.21
Plasma exchange	34.78
Cyclophosphamide	42.39
Rituximab	28.26

**Table 2:** Serum levels of IL-18 and sIL-1Β according to the severity of the kidney interstitial lesions.

	sIL-18 (pg/mL)			sIL-1β (pg/mL)		
	Absent–Mild (Grades 0–1)	Moderate–Severe (Grades 2–3)	*p*-Value	Absent–Mild (Grades 0–1)	Moderate–Severe (Grades 2–3)	*p*-Value
Fibrosis	567.93 ± 599.11	191.34 ± 250.34	0.013	1.36 ± 1.34	0.42 ± 0.59	0.002
Atrophy	474.78 ± 523.43	166.25 ± 268.31	0.062	1.38 ± 1.49	0.2 ± 0.25	0.004
Inflammatory Infiltrate	507.80 ± 513.59	370.27 ± 487.39	0.20	3.27 ± 8.64	0.95 ± 1.29	0.18

**Table 3:** Allelic frequencies of the studied SNPs. MAF, minor allele frequency.

Gene	SNP	Hardy–Weinberg Equilibrium		Minor Allele in the Study Population	MAF in the Study Population	MAF (European Ancestry)
		Chi-Square	*p*-Value			
*IL-1β*	rs1143634	0.237	0.88	A	0.19	A allele, 0.24
*NLRP3*	rs4612666	0.096	0.853	T	0.27	T allele, 0.24
*IL-18*	rs1946518	2.51	0.285	G	0.48	T allele, 0.42
*IL-18*	rs187238	1.152	0.562	G	0.32	G allele, 0.28
*NLRP1*	rs878329	0.509	0.775	C	0.44	C allele, 0.46
*CARD8*	rs2043211	0.182	0.913	T	0.33	T allele, 0.33
*NLRP3*	rs10754558	1.872	0.392	G	0.36	G allele, 0.46
*CASP1*	rs530537	0.163	0.922	C	0.48	C allele, 0.44

**Table 4:** The main analytical variables analyzed and the genotype of the IL-18 SNPs studied. SD, standard deviation; eGFR, estimated glomerular filtration rate; CRP, C-reactive protein; ANCA, anti-neutrophil cytoplasm antibodies.

	*IL-18* rs187238			*IL-18* rs1946518		
	GG n = 7 (7.6%)	C-Carriers n = 85 (92.4%)	*p*-Value	T-Carriers n = 67 (72.8%)	GG n = 25 (27.2%)	*p*-Value
Mean sCreatinine ± SD (µmol/L)	363.68 ± 288.19	206.71 ± 110.35	0.16	360.04 ± 238.12	347.82 ± 296.61	0.90
Mean eGFR ± SD (mL/min)	23.37 ± 18.99	33 ± 21.15	0.20	20.93 ± 18.70	25.17 ± 19.44	0.38
Mean proteinuria ± SD (g/day)	0.78 ± 1.15	0.53 ± 0.78	0.64	0.69 ± 0.58	0.79 ± 1.28	0.75
Mean sCRP ± SD (mg/mL)	68.3 ± 77.98	61.17 ± 73.90	0.83	67.37 ± 66.37	67.80 ± 81.35	0.98
Mean ANCA titer (KarbU/L)	494.58 ± 882.16	469.58 ± 611.73	0.47	809.88 ± 1197.76	371.74 ± 668.78	0.12

**Table 5:** Selected SNPs.

Gene	SNP	Chromosome Location	Functional Consequence	Alleles	MAF (European Ancestry)
*IL-1β*	rs1143634	chr2:112832813	Coding sequence variant, synonymous variant	G/A, transition substitution	A allele, 0.24
*NLRP3*	rs4612666	chr1:247435768	Intron variant	C/T, transition substitution	T allele, 0.24
*IL-18*	rs1946518	chr11:112164735	Upstream transcript variant	T/G, transversion substitution	T allele, 0.42
*IL-18*	rs187238	chr11:112164265	Upstream transcript variant	C/G, transversion substitution	G allele, 0.28
*NLRP1*	rs878329	chr17:5649930	None	G/C, transversion substitution	C allele, 0.46
*CARD8*	rs2043211	chr19:48234449	Stop gained variant	A/T, transversion substitution	T allele, 0.33
*NLRP3*	rs10754558	chr1:247448734	3′UTR variant	C/G, transversion substitution	G allele, 0.46
*CASP1*	rs530537	chr11:105027786	Intron variant	C/T, transition substitution	C allele, 0.44

## Data Availability

The data presented in this study are available upon request from the corresponding author. The data are not publicly available due to privacy and ethical restrictions.

## Supplementary Materials

The following supporting information can be downloaded at: https://www.mdpi.com/article/10.3390/ijms25126479/s1. References [47,48,49,50,51,52,53,54,55,56,57,58,59,60,61,62,63,64,65,66,67,68,69,70,71,72,73,74,75,76] are cited in the Supplementary Materials.
