# Supplementary material for: Evaluating Single-Nucleotide Polymorphisms in Inflammasome Proteins and Serum Levels of IL-18 and IL-1β in Kidney Interstitial Damage in Anti-Neutrophilic Cytoplasmic Antibody-Associated Vasculitis
Source: Int J Mol Sci. 2024 Jun 12;25(12):6479. doi: 10.3390/ijms25126479 (PMC11203640; doi:10.3390/ijms25126479)
Supplement: Supplementary file 1 [file ijms-25-06479-s001.zip › ijms-3018876-supplementary.pdf]

	sIL18 (pg/mL)	*sIL-1β (pg/mL)*	*oIL-1β (pg/mL)*
Female vs Male	387.78±503.44 vs 483.05±444.72	0.91±0.97 vs 3.34±8.66	4.44±10.10 vs 3.30±4.80
Elderly vs young	390.04±380.920 vs 470.17±586.36	2.77±7.33 vs 0.73±0.61	4.49±9.27 vs 2.42±2.84
ANCA MPO vs PR3	377.46±442.21 vs 583.79±578.31	1.01±1.12 vs 4.71±11.26	2.69±4.02 vs 8.50±15.69
Crescentic vs other	472.24±545.91 vs 400.46±482.76	4.19±10.75 vs 1.06±1.32	3.55±4.34 vs 3.79±9.25
Esclerotic vs other	75.82±65.90 vs 458.47±507.29*	0.19±0.32 vs 2.11±6.02*	1.13±1.96 vs 4.02±8.55
Mixed vs other	416.83±469.51 vs 430.55±491.69	0.94±0.79 vs 2.42±6.95	5.11±11.79 vs 3.15±4.41
Focal vs other	515.65±576.72 vs 401.57±455.29	1.67±2.02 vs 1.92±6.16	2.26±3.62 vs 4.29±8.73

Supplementary table S1. Supplementary table S1 shows levels of IL-18 and IL-1β in serum and urinary IL-1β, and the comparison of these values according to sex, age (elderly patients were considered as those above 65 years old), ANCA specificity and Berden Histopathological classification. * $p<0.05$

	IL-1β rs1143634				NLRP1 rs878329				CARD8 rs2043211				NLRP3 rs10754558				NLRP3 rs4612666				CASP1 rs530537			
	AA	GA	AA	p-value	CC	CG	GG	p value	AA	AT	TT	p value	CC	CG	GG	p value	CC	CT	TT	p value	CC	CT	TT	p value
Moderate-severe Tubular atrophy (% patients)	100	42.1	27.8	0.08	30.8	42.9	20	0.31	38.5	34.6	20	0.73	31.8	38.5	25	0.75	34.5	37.5	25	0.88	42.9	33.3	30.8	0.77
Moderate-severe Interstitial fibrosis (% patients)	50	41.7	42.6	0.97	27.8	50	38.9	0.28	41.2	46.9	28.6	0.66	45.2	44.8	25	0.44	48.7	33.3	50	0.43	52.9	40	38.1	0.61
Moderate-severe Interstitial infiltrate (% patients)	100	55.6	61.7	0.32	68.4	58.3	55	0.67	64.9	56.3	62.5	0.76	58.8	70	41.7	0.23	62.5	56.7	83	0.47	68.4	59.5	57.1	0.74
Mean sCreat±SD (µmol/L)	504.75± 194.47	395.11± 344.74	320.16± 250.32	0.29	398± 398.78	327.85± 239.97	344.59± 255.7	0.68	333.30± 229.75	406.58± 350.11	233.90± 130.17	0.18	371.79± 307.62	366.10± 290.57	249.73± 159.54	0.33	376.62± 304.72	326.80± 271.86	317.67± 159.82	0.71	362.86± 261.79	344.86± 303.77	351.58± 266.56	0.97
Mean eGFR±SD (ml/min)	10.83± 5.39	24.64± 17.85	24.08± 19.23	0.48	28.09± 22.94	23.61± 19.27	23.15± 16.89	0.67	23.81± 17.77	23.77± 21.12	26.19± 19.44	0.94	22.77± 18.91	24.27± 20.96	28.12± 16.72	0.67	23.61± 20.26	25.40± 19.84	19.3± 6.38	0.77	23.42± 18.11	23.77± 18.05	23.20± 22.81	0.95
Mean proteinuria±SD (g/day)	0.33± 0.03	1.14± 1.69	0.76± 1.12	0.14	1.01± 1.83	0.68± 0.69	0.75± 1.05	0.67	1.12± 1.15	0.56± 0.65	0.31± 0.21	0.07	0.71± 0.94	0.59± 0.55	1.18± 1.96	0.34	0.82± 1.29	0.57± 0.61	2.14± 1.99	0.07	0.56± 0.67	0.77± 1.31	0.92± 1.13	0.66
Mean sCRP±SD (mg/mL)	56.95± 42.639	80.76± 81.73	60.76± 75.96	0.58	78.27± 95.35	65.69± 79.96	63.97± 63.81	0.85	58.65± 69.74	76.42± 87.97	71.33± 70.24	0.67	75.35± 84.55	78.40± 77.96	29.28± 38.33	0.15	74.5± 88.29	63.1±64.41	27.37± 20.48	0.55	49.62± 60.51	67.77± 73.68	83.07± 93.67	0.41
Mean ANCA titer (KarbU/L)	151.77± 123.56	581.68± 1169.58	471.01± 715.59	0.69	545.02± 740.48	565.87± 1093.24	303.74± 419.99	0.49	553.18± 986.47	470.01± 815.88	351.86± 514.39	0.78	580.65± 945.97	483.07± 854.62	340.14± 727.28	0.67	467.96± 764.09	438.04± 980.76	941.42± 884.73	0.48	826.19± 1469.39	435.50± 606.91	322.15± 480.11	0.16
Death within the first year after diagnosis (%patients)	0	3.6	3.3	0.93	0	2.4	3.3	0.74	4.8	2.6	0	0.7	5	2.8	0	0.64	2.1	5.4	0	0.63	0	4.5	3.8	0.61
Relapse within the first year after diagnosis (%patients)	0	11.1	15.3	0.63	21.1	12.5	6.9	0.35	19.5	5.3	18.2	0.16	18.4	8.3	13.3	0.45	14.9	10.8	20	0.78	18.2	14.3	7.7	0.55

Supplementary table S2. Supplementary table S2 shows the interstitial lesions found in kidney biopsy at diagnosis, the analytical parameters evaluated and disease outcomes according to the different genotypes of IL-1β rs1143634, NLRP1 rs878329, CARD8 rs2043211, NLRP3 rs10754558, NLRP3 rs4612666, CASP1 rs530537.

GENE	dbSNP	Functional consequence	MAF	References of studies related to autoimmune disease
NLRP 3	Rs4612666	Intron variant	0.24	[47]: ulcerative colitis
	Rs10754558	3'UTR variant	0.46	[47-51]: rheumatoid arthritis, ulcerative colitis, celiac disease, type-1 diabetes
	Rs35829419	Stop gained variant	0.05	-
	Rs3806265	Synonymous variant	0.33	[47]: ulcerative colitis
	Rs3806268	Silent variant	0.45	-
NLRP 1	Rs878329	None	0.46	[52-54]: Vogt-Koyanagi-Harada disease, rheumatoid arthritis, ankylosing spondylitis, rheumatoid arthritis
	Rs2670660	None	0.46	[49, 55-58]: vitiligo, thyroid disease, lupus, celiac disease
	Rs6502867	Intron variant	0.25	[54,55,57,52]: vitiligo, Vogt-Koyanagi-Harada disease, ankylosing spondylitis
	Rs4790797	None	0.46	[55]
	Rs8182352	None	0.39	[55]
MEFV	Rs224204	Intron variant	0.48	-
CASP 1	Rs530537	Intron variant	0.44	[62]
	Rs568910	Intron variant	0.17	-
	Rs580253	Synonymous variant	0.17	-
CARD 8	Rs2043211	Stop gained variant	0.33	[51, 60-64]: type-1 diabetes, celiac disease, rheumatoid arthritis, gout, Crohn's disease
	Rs11672725	Intron variant	0.18	-
IL-1B	RS1143634	Coding synonymous variant	0.24	[65-70]: lupus erythematosus, systemic sclerosis, Kawasaki disease, Bullous Pemphigoid
IL-18	Rs1946518	Upstream transcript variant	0.42	[71-74]: rheumatoid arthritis, type-1 diabetes, kidney transplant
	Rs187238	Upstream transcript variant	0.28	[71-76]: rheumatoid arthritis, type-1 diabetes.

Supplementary table S3. Detailed results of the literature research and selection of SNPs.

47. Hanaei S, Sadr M, Rezaei A, et al. Association of NLRP3 single nucleotide polymorphisms with ulcerative colitis: A case-control study. *Clin Res Hepatol Gastroenterol*. 2018;42(3):269-275. doi:10.1016/j.clinre.2017.09.003
48. Pontillo A, Brandao L, Guimaraes R, Segat L, Araujo J, Crovella S. Two SNPs in NLRP3 gene are involved in the predisposition to type-1 diabetes and celiac disease in a pediatric population from northeast Brazil. *Autoimmunity*. 2010;43(8):583-589. doi:10.3109/08916930903540432

49. Pontillo A, Vendramin A, Catamo E, Fabris A, Crovella S. The missense variation Q705K in CIAS1/NALP3/NLRP3 gene and an NLRP1 haplotype are associated with celiac disease. *Am J Gastroenterol*. 2011;106(3):539-544. doi:10.1038/ajg.2010.474
50. Lee YH, Bae SC. Association between functional NLRP3 polymorphisms and susceptibility to autoimmune and inflammatory diseases: a meta-analysis. *Lupus*. 2016;25(14):1558-1566. doi:10.1177/0961203316644336
51. Addobbati C, da Cruz HLA, Adelino JE, et al. Polymorphisms and expression of inflammasome genes are associated with the development and severity of rheumatoid arthritis in Brazilian patients. *Inflamm Res*. 2018;67(3):255-264. doi:10.1007/s00011-017-1119-2
52. Horie Y, Saito W, Kitaichi N, Miura T, Ishida S, Ohno S. Evaluation of NLRP1 gene polymorphisms in Vogt-Koyanagi-Harada disease. *Jpn J Ophthalmol*. 2011;55(1):57-61. doi:10.1007/s10384-010-0887-9
53. Sui J, Li H, Fang Y, et al. NLRP1 gene polymorphism influences gene transcription and is a risk factor for rheumatoid arthritis in han chinese. *Arthritis Rheum*. 2012;64(3):647-654. doi:10.1002/art.33370
54. Sun R, Huang Y, Zhang H, Liu R. MMP-2, TNF-α and NLRP1 polymorphisms in Chinese patients with ankylosing spondylitis and rheumatoid arthritis. *Mol Biol Rep*. 2013;40(11):6303-6308. doi:10.1007/s11033-013-2743-8
55. Dieudé P, Guedj M, Wipff J, et al. NLRP1 influences the systemic sclerosis phenotype: a new clue for the contribution of innate immunity in systemic sclerosis-related fibrosing alveolitis pathogenesis. *Ann Rheum Dis*. 2011;70(4):668-674. doi:10.1136/ard.2010.131243
56. Pontillo A, Girardelli M, Kamada AJ, et al. Polymorphisms in inflammasome genes are involved in the predisposition to systemic lupus erythematosus. *Autoimmunity*. 2012;45(4):271-278. doi:10.3109/08916934.2011.637532
57. Dwivedi M, Laddha NC, Mansuri MS, Marfatia YS, Begum R. Association of NLRP1 genetic variants and mRNA overexpression with generalized vitiligo and disease activity in a Gujarat population. *Br J Dermatol*. 2013;169(5):1114-1125. doi:10.1111/bjd.12467
58. Rajendiran KS, Rajappa M, Chandrashekar L, Thappa DM. Association of Nod-like receptor protein-1 (rs2670660) and Toll-like receptor-4 (rs4986790) with non-segmental vitiligo: A case-control study in South Indian population. *Int J Immunogenet*. 2019;46(5):321-330. doi:10.1111/iji.12429
59. Sharab LY, Morford LA, Dempsey J, et al. Genetic and treatment-related risk factors associated with external apical root resorption (EARR) concurrent with orthodontia. *Orthod Craniofac Res*. 2015;18 Suppl 1(Suppl 1):71-82. doi:10.1111/ocr.12078
60. Ben Hamad M, Cornelis F, Marzouk S, et al. Association study of CARD8 (p.C10X) and NLRP3 (p.Q705K) variants with rheumatoid arthritis in French and Tunisian populations. *Int J Immunogenet*. 2012;39(2):131-136. doi:10.1111/j.1744-313X.2011.01070.x

61. Yi M, Shao X, Ma J, Tian B, Zhang Y, Liu S. rs2043211 polymorphism in CARD8 is not associated with Tourette syndrome in a family-based association study in the Chinese Han population. *Int J Psychiatry Med*. 2015;49(3):208-214. doi:10.1177/0091217415582190
62. Zhang ZT, Ma XJ, Zong Y, Du XM, Hu JH, Lu GC. Is the CARD8 rs2043211 polymorphism associated with susceptibility to Crohn's disease? A meta-analysis. *Autoimmunity*. 2015;48(8):524-531. doi:10.3109/08916934.2015.1045581
63. Lee SW, Lee SS, Oh DH, et al. Genetic Association for P2X7R rs3751142 and CARD8 rs2043211 Polymorphisms for Susceptibility of Gout in Korean Men: Multi-Center Study. *J Korean Med Sci*. 2016;31(10):1566-1570. doi:10.3346/jkms.2016.31.10.1566
64. Smigoc Schweiger D, Goricar K, Hovnik T, et al. Dual Role of *PTPN22* but Not *NLRP3* Inflammasome Polymorphisms in Type 1 Diabetes and Celiac Disease in Children. *Front Pediatr*. 2019;7:63. Published 2019 Mar 12. doi:10.3389/fped.2019.00063
65. Tahmasebi Z, Akbarian M, Mirkazemi S, et al. Interleukin-1 gene cluster and IL-1 receptor polymorphisms in Iranian patients with systemic lupus erythematosus. *Rheumatol Int*. 2013;33(10):2591-2596. doi:10.1007/s00296-013-2784-2
66. Abtahi S, Farazmand A, Mahmoudi M, et al. IL-1A rs1800587, IL-1B rs1143634 and IL-1R1 rs2234650 polymorphisms in Iranian patients with systemic sclerosis. *Int J Immunogenet*. 2015;42(6):423-427. doi:10.1111/iji.12212
67. Kapelski P, Skibinska M, Maciukiewicz M, et al. Association study of functional polymorphisms in interleukins and interleukin receptors genes: IL1A, IL1B, IL1RN, IL6, IL6R, IL10, IL10RA and TGFB1 in schizophrenia in Polish population. *Schizophr Res*. 2015;169(1-3):1-9. doi:10.1016/j.schres.2015.10.008
68. Assari R, Aghighi Y, Ziaee V, et al. Pro-inflammatory cytokine single nucleotide polymorphisms in Kawasaki disease. *Int J Rheum Dis*. 2018;21(5):1120-1126. doi:10.1111/1756-185X.12911
69. Shehjar F, Afroze D, Misgar RA, Malik SA, Laway BA. Association of polymorphic variants of IL-1β and IL-1RN genes in the development of Graves' disease in Kashmiri population (North India). *Hum Immunol*. 2018;79(4):228-232. doi:10.1016/j.humimm.2018.02.006
70. Tabatabaei-Panah PS, Moravvej H, Sadaf Z, et al. Proinflammatory Cytokine Gene Polymorphisms in Bullous Pemphigoid. *Front Immunol*. 2019;10:636. Published 2019 Mar 29. doi:10.3389/fimmu.2019.00636
71. Pawlik A, Kurzawski M, Drozdzik M, Dziedziejko V, Safranow K, Herczynska M. Interleukin-18 gene (IL18) promoter polymorphisms in patients with rheumatoid arthritis. *Scand J Rheumatol*. 2009;38(3):159-165. doi:10.1080/03009740802600748
72. Tavares NA, Santos MM, Moura R, et al. Interleukin 18 (IL18) gene promoter polymorphisms are associated with type 1 diabetes mellitus in Brazilian patients. *Cytokine*. 2013;62(2):286-289. doi:10.1016/j.cyto.2013.03.004

73. do Nascimento WG, Cilião DA, Genre J, et al. Genetic polymorphisms of Interleukin-18 are not associated with allograft function in kidney transplant recipients. *Genet Mol Biol*. 2014;37(2):343-349. doi:10.1590/s1415-47572014005000005
74. Celik SD, Ates O. Genetic analysis of interleukin 18 gene polymorphisms in alopecia areata. *J Clin Lab Anal*. 2018;32(5):e22386. doi:10.1002/jcla.22386
75. Messaoudi S, Dandana M, Magdoud K, et al. Interleukin-18 promoter polymorphisms and risk of idiopathic recurrent pregnancy loss in a Tunisian population. *J Reprod Immunol*. 2012;93(2):109-113. doi:10.1016/j.jri.2011.12.002
76. Al-Lahham Y, Mendes AKB, Souza EM, et al. Interleukin-18 (rs187238) and glucose transporter 4 (rs5435) polymorphisms in Euro-Brazilians with type 1 diabetes. *Genet Mol Res*. 2017;16(3):10.4238/gmr16039755. Published 2017 Sep 21. doi:10.4238/gmr16039755
